# Human Novelty Response to Emotional Animal Vocalizations: Effects of Phylogeny and Familiarity

**DOI:** 10.3389/fnbeh.2017.00204

**Published:** 2017-10-24

**Authors:** Marina Scheumann, Anna S. Hasting, Elke Zimmermann, Sonja A. Kotz

**Affiliations:** ^1^Institute of Zoology, University of Veterinary Medicine Hannover, Hannover, Germany; ^2^Neuropsychology, Max Planck Institute for Human Cognitive and Brain Sciences, Leipzig, Germany; ^3^Day Clinic for Cognitive Neurology, University Hospital Leipzig, Leipzig, Germany; ^4^Faculty of Psychology and Neuroscience, Maastricht University, Maastricht, Netherlands

**Keywords:** auditory ERP, novelty oddball, sound familiarity, emotion processing, voice, phylogeny

## Abstract

Darwin (1872) postulated that emotional expressions contain universals that are retained across species. We recently showed that human rating responses were strongly affected by a listener's familiarity with vocalization types, whereas evidence for universal cross-taxa emotion recognition was limited. To disentangle the impact of evolutionarily retained mechanisms (phylogeny) and experience-driven cognitive processes (familiarity), we compared the temporal unfolding of event-related potentials (ERPs) in response to agonistic and affiliative vocalizations expressed by humans and three animal species. Using an auditory oddball novelty paradigm, ERPs were recorded in response to task-irrelevant novel sounds, comprising vocalizations varying in their degree of phylogenetic relationship and familiarity to humans. Vocalizations were recorded in affiliative and agonistic contexts. Offline, participants rated the vocalizations for valence, arousal, and familiarity. Correlation analyses revealed a significant correlation between a posteriorly distributed early negativity and arousal ratings. More specifically, a contextual category effect of this negativity was observed for human infant and chimpanzee vocalizations but absent for other species vocalizations. Further, a significant correlation between the later and more posteriorly P3a and P3b responses and familiarity ratings indicates a link between familiarity and attentional processing. A contextual category effect of the P3b was observed for the less familiar chimpanzee and tree shrew vocalizations. Taken together, these findings suggest that early negative ERP responses to agonistic and affiliative vocalizations may be influenced by evolutionary retained mechanisms, whereas the later orienting of attention (positive ERPs) may mainly be modulated by the prior experience.

## Introduction

The recognition of emotions conveyed in the human voice plays an important role in human social interactions. Humans can decode prosodic cues related to the emotional state of the sender from human speech and non-linguistic vocalizations (e.g., Zeskind and Marshall, 1988; Fecteau et al., 2005; Sander et al., 2007; Belin et al., 2008; Jessen and Kotz, 2011; Ho et al., 2015; Kokinous et al., 2015). Cross-cultural studies indicate a universal pattern in the expression and perception of these prosodic cues (e.g., Scherer et al., 2001; Juslin and Laukka, 2003; Pell et al., 2009a,b; Sauter et al., 2010; Liu et al., 2015) and suggest a pre-human origin predominantly organized by innate mechanisms.

More than 130 years ago Darwin (1872) postulated in his masterpiece “The Expression of Emotion in Man and Animals” that emotional expressions contain universals that are retained across mammalian species by evolutionary mechanisms. Inspired by Darwin, Morton (1977) compared agonistic, fearful, and affiliative vocalizations across mammals and birds and proposed the so-called motivation-structural rules. Thus, in mammals relatively low frequency and broadband (harsh) sounds are associated with aggressive contextual behavior, whereas high frequency sounds with a tonal structure are associated with fearful or friendly contextual behavior. Ehret (2006) extended this model of Morton (1977) and suggested that the perception of communicative sounds of mammals conveys three basic meanings: (1) aversion, (2) attraction, and (3) cohesion. Calls inducing aversion should cover a broad frequency range of a varying frequency spectrum with noisy components. Calls attracting the recipient should be high frequency tonal sounds, whereas calls inducing cohesion should be associated with a low frequency rhythmic structure. To date, various empirical investigations support the idea of cross-taxa similarities in emotional vocalizations across different mammalian groups (e.g., Soltis et al., 2005; Scheumann et al., 2007, 2012; Bastian and Schmidt, 2008; Schehka and Zimmermann, 2009; Gogoleva et al., 2010; Zimmermann, 2010). Thereby, the encoding of acoustically conveyed emotion in animal vocalizations show similarities with prosodic cues in human vocalizations and speech (e.g., Vettin and Todt, 2005; Hammerschmidt and Jürgens, 2007; Davila Ross et al., 2009; Zimmermann et al., 2013). These results are further supported by playback studies on cross-taxa recognition. These studies already showed that humans are able to classify context and valence-specific animal vocalizations (cats: Nicastro and Owren, 2003; dogs: Pongrácz et al., 2005, 2006, 2010, 2011; Molnár et al., 2006, 2010; Flom et al., 2009; Taylor et al., 2009; pigs: Tallet et al., 2010; macaques: Linnankoski et al., 1994). However, in most of these studies human participants only listened to one species, either a phylogenetically closely related species (primates) or a somewhat familiar species (domesticated species e.g., dog, cats). Thus, it remains unclear whether recognizing emotional vocalizations across species can be explained by cross-taxa universal coding and processing mechanisms as a result of phylogeny or by familiarity alone.

In a previous study (Scheumann et al., 2014), we investigated vocally induced cross-taxa emotion recognition by using agonistic and affiliative vocalizations of human infants (conspecific control) and three animal species varying in their degree of familiarity and phylogeny to humans. This was done to explain the effect of familiarity and phylogeny on vocally induced cross-taxa emotion recognition. We found that adult human male listeners showed the highest emotion recognition accuracy for conspecific vocalizations, while the recognition accuracy for animal vocalizations was mainly dependent on call type familiarity, i.e., the recognition of species-specific vocalization types/context. These findings suggest that at least in an explicit task, cross-taxa vocalization-induced emotion recognition in adult male listeners is more affected by cognitive experience-based mechanisms than by phylogeny. This finding also aligns with Belin et al. (2008), who reported that humans are unable to recognize the valence of animal vocalizations using an explicit behavioral rating task. Interestingly, fMRI data collected in parallel, revealed brain activation in the right ventro-lateral orbitofrontal cortex (OFC) in response to negative and positive vocalizations of humans, non-human primates (rhesus monkey), and non-primates (domestic cat). The authors explained the discrepancy between the behavioral rating and the imaging results by an unconscious evolutionary retained brain mechanism differentiating the valence of human and animal vocalizations. This mechanism may be masked by later cognitive processes in explicit behavioral rating tasks. Although, Belin et al. (2008) argued that the shared systems underlying emotion processing may engage at an unconscious level, any conclusions about an automatic response to cross-taxa vocalizations in an explicit behavioral task would be complicated by specific task demands. Furthermore, it may be argued that evolutionary hard-wired and therefore rather automatic brain responses to emotional vocalizations act on a different time scale than higher-order cognitive ones, which calls for a more time-sensitive method than functional magnetic resonance imaging (fMRI). Consequently, to disentangle the respective impact of evolutionarily retained mechanisms (i.e., phylogeny) and experience-driven cognitive processes (i.e., familiarity) on emotional processing in humans, a time-sensitive implicit approach is needed.

To fill this gap, we performed an ERP experiment to explore the temporal dynamics of cross-taxa agonistic and affiliative vocalizations that varied in their degree of phylogenetic relatedness and familiarity to humans. We employed a widely used ERP paradigm, the auditory novelty oddball paradigm that allows the parallel testing of novelty attentional orienting as well as familiarization of novel auditory sounds (e.g., Friedman et al., 2001). This classical ERP paradigm includes task-relevant standard and deviant tones (targets) as well as task-irrelevant novel sounds (= novels, in the current case the emotional contextual category). Using this paradigm, Czigler et al. (2007) reported a biphasic ERP response of an early negativity and a late positivity comparing aversive versus neutral novels. In particular, the early negative response to aversive novels may be explained by their relevance for an organisms' survival (e.g., Sauter and Eimer, 2010; Schirmer and Escoffier, 2010) as aversive sounds may lead to negative consequences for the organism. Czigler et al. (2007) suggested that the early negative response may engage a broader neural network including the limbic system and the auditory cortex whereas the late positivity may reflect cognitive evaluation. Early emotional processing of prosodic cues around 100–200 ms after stimulus onset have also been reported in human speech (e.g, Schirmer et al., 2005; Schirmer and Kotz, 2006; Paulmann and Kotz, 2008; Paulmann et al., 2013) and non-linguistic vocalizations (Sauter and Eimer, 2010; Jessen and Kotz, 2011; Liu et al., 2012; Pell et al., 2015). Thus, early ERPs seem ideally suited for studying possible evolutionary retained mechanism underlying the cross-specific perception of emotional vocalizations.

Investigating the effect of familiarity on auditory processing, studies reported early ERP responses (e.g., N1, MMN) that do not have to engage attention to the stimulus dimension (Shahin et al., 2003, 2004; Thierry et al., 2003; Jacobsen et al., 2005; Kirmse et al., 2009, 2012) and higher-order cognitive processes related to later positivities such as the P3 complex (e.g., Cycowicz and Friedman, 1998; Kirmse et al., 2009). Thus, larger N1 and MMN responses to familiar than unfamiliar sounds were found (Thierry et al., 2003; Kirmse et al., 2012), and an increased novelty P3 responses to unfamiliar stimuli (Cycowicz and Friedman, 1998).

The current study utilized the auditory novelty oddball paradigm to compare the novelty response of agonistic and affiliative vocalizations of different species (human infants, dogs, chimpanzees, and tree shrews) varying in their degree of phylogeny and familiarity with respect to humans. Based on previous behavioral and ERP findings, the following hypotheses were derived for the present study:

1. As phylogeny is likely to play an important role in early ERP responses to vocalizations expressed in agonistic contexts due to their high relevance for an organisms' survival, we expected an enhanced early negative ERP response to agonistic human infant and chimpanzee vocalizations (phylogenetically closely related species) compared to vocalizations produced in affiliative contexts. We did not expect these early ERPs for dog or tree shrew vocalizations (phylogenetically far related species).
2. As ERP familiarity effects have been shown both early and later, we expected ERP responses to vary as a function of familiarity ratings given by the participants after the ERP experiment. Thus, novel contextual categories, which were rated as more familiar were expected to increase ERP responses compared to unfamiliar novel categories. ERP responses were also correlated with the participants' offline stimulus ratings of valence, arousal, and familiarity to specify their functional significance as well as to acoustic dissimilarities between standard and novels to control for mere acoustic effects.

## Materials and methods

### Participants

Thirty male participants took part in the experiment to circumvent gender specific responses to emotional vocalizations (e.g., Schirmer et al., 2004, 2005) and especially infant vocalizations (e.g., Seifritz et al., 2003; Sander et al., 2007). The age range of participants was 21–28 years (mean = 24 years, *SD* = 2), all were right handed (LQ: mean = 51.5, *SD* = 13.7) according to an abbreviated version of the Edinburgh Inventory (Oldfield, 1971), and self-reported no hearing or neurological deficits. To avoid ceiling effects of familiarity, no participant had children or owned a dog. They received 7 Euros per hour for their participation. The study was approved by the Ethics committee of the University of Leipzig and was conducted in concordance with the Declaration of Helsinki.

### Acoustic stimuli

Standard tones were sinusoidal tones of 600 Hz frequency and 756 ms duration (including 10 ms rise and fall and the duration matched to the mean duration of the novel sounds). Target (deviant) tones differed from the standard tones in frequency only (660 Hz). Novel sounds were vocalizations of four different species (human infant, dog, chimpanzee, and tree shrew), recorded in two emotionally distinct behavioral context categories (affiliative and agonistic). These stimuli were identical to the ones of our behavioral rating study (Scheumann et al., 2014) where more detailed information on the recording context and the stimulus preparation can be found (but see also Supplementary document paragraphs 1, 2, and 3). For each of the species and context categories 24 stimuli were selected from recordings of 5 to 8 different senders, resulting in a total of 192 vocalizations grouped in eight categories: agonistic human infant, affiliative human infant, agonistic dog, affiliative dog, agonistic chimpanzee, affiliative chimpanzee, agonistic tree shrew, affiliative tree shrew. All stimuli were sampled at 44.1 kHz (16 bit, mono). Sound intensity was normalized to 60 dB using PRAAT (www.praat.org; Boersma, 2001).

### Design and experimental procedure

In the auditory novelty oddball paradigm, participants listened to continuous sequences of auditory events. Seventy-six percent of these events were standard tones, 12% were target tones, and 12% were novel sounds. Together, the standards and targets formed the auditory scene that was task relevant. The novels were task-irrelevant and non-repetitive. The sequence was pseudo-randomized for each participant with the following constraints: (i) at least three events occurred between consecutive novels or targets, (ii) targets and novels were not allowed in direct succession, and (iii) a maximum of two targets or two novels occurred before the next occurrence of the next deviant event. The stimuli were presented at an inter-stimulus-interval (ISI) of 756 ms to maximize isochrony and hence the signal-to-noise ratio. The entire sequence of 1,600 acoustic events (1,216 standards, 192 targets, 192 novels) was divided into four blocks with slightly different numbers of targets (46, 56, 47, 43).

For the duration of the experiment, participants sat in an acoustically and electrically shielded chamber in a comfortable chair. Acoustic stimulation was administered via headphones (Audio-Technica ATH-M40fs). Participants were instructed to attend to the sequences and to silently count the number of deviant tones in each block while fixating a cross that was presented continuously on a computer screen positioned about 140 cm in front of them. They were asked to move, swallow, or blink as little as possible during the auditory stimulation. The first block was preceded by 11 events (9 standards, 2 targets) to initiate the task (thus participants encountered 48 targets in the first block). These were not included in the analysis. Between blocks, participants were asked for the deviant count and were allowed to take short breaks to move and rest their eyes. The duration of the experiment was ~40 min excluding breaks and EEG setup-time.

### EEG recording and analysis

Continuous EEG signals were recorded from 61 Ag/AgCl electrodes mounted in an elastic cap (Electro-Cap International). The locations of the electrodes were: FPz, FP1/2, AFz, AF1/2, AF3/4, AF7/8, Fz, F3/4, F5/6, F7/8, F9/10, FCz, FC3/4, FC5/6, FT7/8, FT9/10, Cz, C1/2, C3/4, C5/6, T7/8, CPz, CP3/4, CP5/6, TP7/8, TP9/10, Pz, P3/4, P5/6, P7/8, P9/10, POz, PO3/4, PO7/8, Oz, O1/2 according to the nomenclature proposed by the American Electroencephalographic Society (Sharbrough et al., 1991). Additional electrodes were placed at the mastoids (A1 and A2). The ground electrode was located at the sternum. To control for ocular artifacts, bipolar horizontal and vertical electrooculograms (HEOG and VEOG) were recorded from the outer canthus of each eye and from above and below the right eye, respectively. The mean of all electrodes served as on-line reference. Electrodes were connected to a Refa amplifier (Twente Medical Systems, The Netherlands). Signals were sampled on-line at a rate of 500 Hz (DC to 135 Hz, anti-aliasing filter). Electrode impedances were kept below 5 kΩ throughout the whole experiment.

Offline, data pre-processing and ERP analyses were performed using the EEP 3.2 software package (Max-Planck institute of Human Cognitive and Brain Sciences, Leipzig, commercially available as EEProbe, ANT Neuro). Epochs of −100 to 800 ms with respect to the stimulus onsets were selected and scanned semi-automatically for artifacts. Epochs with a voltage variation of more than 40 μV on VEOG or Cz or 30 μV on HEOG within a 200 ms sliding time window were marked as contaminated by artifacts. Contaminated epochs containing eye blinks or saccades identified via electrooculography were used to obtain propagation factors which were calculated on the basis of 30 prototypical blinks and 30 prototypical moves selected separately for each participant. The propagation factors were then used to compensate for prototypical artifacts via a regression algorithm (Electrooculogram Epoch Classification), implemented in the EEP software (see also Pfeifer et al., 1995). Artifact-free or—corrected epochs were averaged separately for each participant and stimulus type (standards, targets, novels) after error trials or time-outs (<2% in all conditions) were removed. Additionally, an average for each of the eight novel categories was created per participant. The 100 ms prior to the onset served as baseline. The averaged data were re-referenced to the average of both mastoid channels and used for to calculate means for each participant and each ERP component. For presentation purposes only the data were filtered at 14 Hz low-pass.

**Figure 1:**
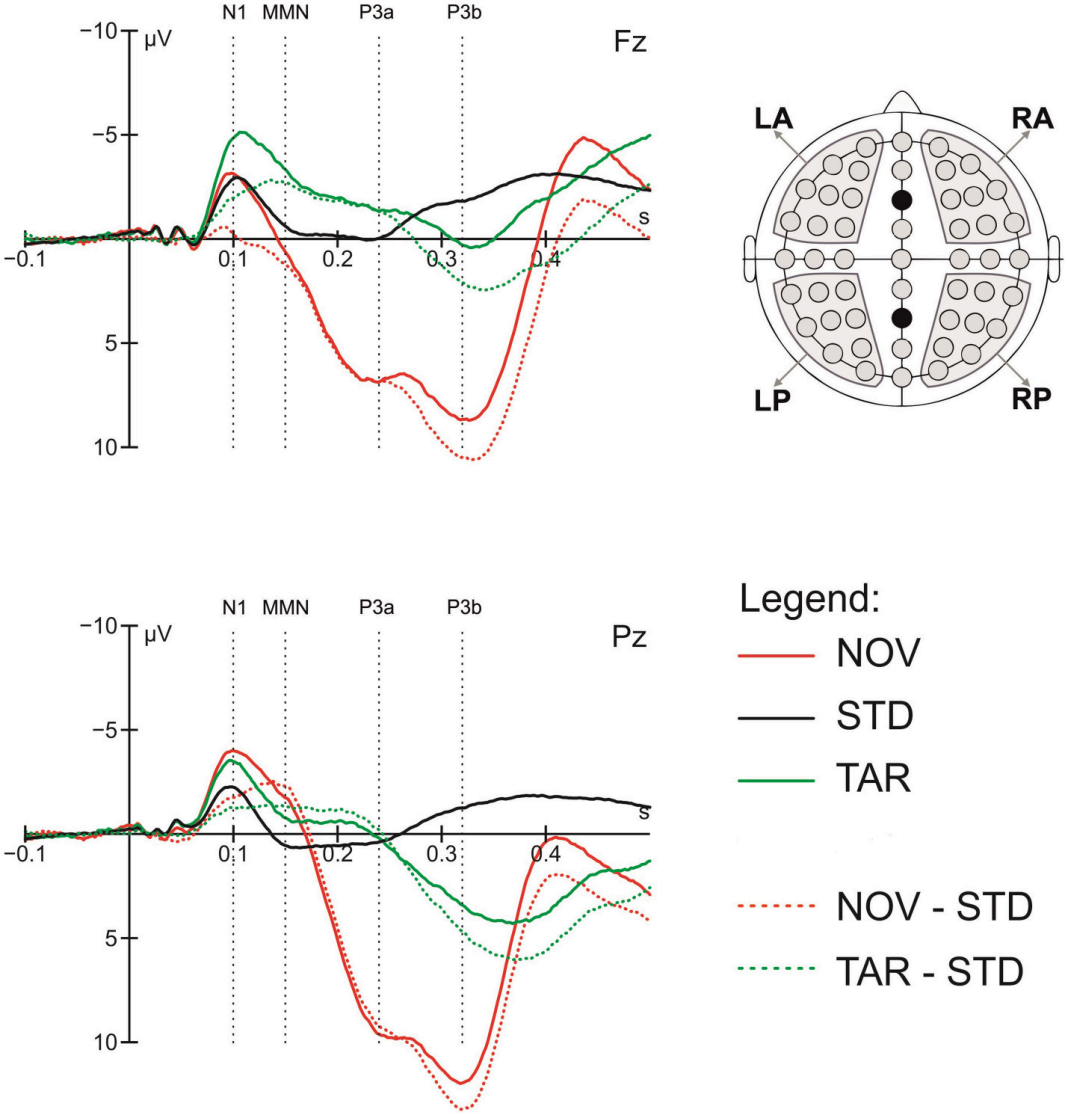
Grand average ERP response to standards (STD), targets (TAR) and novels (NOV), novel-standard (NOV-STD), and target-standard (NOV-TAR) difference waves at the Fz and Pz electrode and component definition; the head on the right shows regions of interest (ROIs) used in the statistical analysis; dotted lines show center of the 60 ms time windows for the N1, MMN, P3a, and P3b component.

To quantify the ERP components of interest, the general target-detection and novelty responses were first assessed by comparing the ERPs in response to target tones and novel sounds to those to standard tones, respectively. Based on visual inspection of the grand average data, time windows of 60 ms were centered on visible peaks or time points of largest differences between conditions for the following components: N1 (70–130 ms), MMN (120–180 ms), P3a (210–270 ms), and P3b (290–350 ms). The latency of the ERP components was measured relative to stimulus onset. To validate the data, we pre-analyzed ERPs to deviants and novels, which showed the typical pattern of an orienting response comprising N1, MMN, P3a, and P3b components (Friedman et al., 2001). Figure 1 shows the grand average ERP peak latency responses to standard tones, deviant tones, and novel sounds in these time windows.

### Acoustic analyses of vocalizations

Acoustic analyses were performed for all novel sounds measuring two spectral parameters (center of gravity, mean peak frequency), a tonality-related parameter (percentage of voiced frames) and an amplitude-related parameter (percentage of call energy) using PRAAT (see Table S1). To account for the fact that ERPs reflect sound processing in real time, we adapted acoustic analyses from the start of the stimuli to the onset of the ERPs of interest. Thus, the measurements reflect the acoustical properties of the novels up to the time point where the analyzed time window of the ERP component starts. Thus, we measured the acoustic parameters for four intervals: from stimulus onset to 70 ms (N1), from stimulus onset to 120 ms (MMN), from stimulus onset to 210 ms (P3a), and from stimulus onset to 290 ms (P3b) after stimulus onset.

### Rating study

Four to six weeks after the EEG recording, 28 of the initial participants returned for a behavioral rating study. We choose this long period to reduce the possibility that participants remember the stimuli from the previous EEG session. Participants listened to the calls again, in the same order, in which they had been presented to them in the EEG. The major results of this rating study were already published in Scheumann et al. (2014). Here, we will use the quantitative ratings for emotional valence and arousal (referring to the ratings of self-perspective in Scheumann et al., 2014) and the familiarity (referring to the assumed familiarity rating in Scheumann et al., 2014) for correlational analyses with the expected ERP responses. The ratings of emotional valence and arousal were based on five point versions of the self-assessment manikin (SAM; Bradley and Lang, 1994). For familiarity, participants rated the vocalizations on a scale ranging from “not familiar” (1) to “very familiar” (5).

### Statistical analysis

To assess topographical differences and to reduce α-error accumulation by multiple testing of single electrodes, electrodes were grouped into four regions of interest (ROIs) as representatives of the topographical distribution: left anterior (LA: FP1, AF7, AF3, F7, F5, F3, FT7, FC5, FC3), left posterior (LP: TP7, CP5, CP3, P7, P5, P3, PO7, PO3, O1), right anterior (RA: FP2, AF4, AF8, F4, F6, F8, FC4, FC6, FT8), and right posterior (RP: CP4, CP6, TP8, P4, P6, P8, PO4, PO8, O2). The mean amplitudes across electrodes were calculated for each of the ROIs and subjected to statistical analysis.

To analyse the ERP responses to novels, a 4 × 2 × 2 × 2 repeated-measures ANOVA was calculated based on the mean amplitude for each participant and time window, using the factors species (SPEC; human infant, chimpanzee, dog, tree shrew), context (CON; affiliative/ agonistic), and the two topographical factors: region (REG; anterior/posterior), and hemisphere (HEM; right/left). If the Mauchly's test indicated that the assumptions of sphericity are violated (*p* ≤ 0.05), we corrected the degrees of freedom using Greenhouse-Geisser estimates of sphericity (Field, 2009). Consistent with our previous findings (Scheumann et al., 2014), data showed interactions between SPEC and CON, thus we conducted a step-down analysis for each species separately, i.e., 2 × 2 × 2 repeated measurement ANOVAs using the factors CON, HEM, and REG. If the factor CON showed an interaction with one of the topographical factors, we performed a further step-down analysis comparing context categories for ROI using the Hotelling *T*-Test.

To specify the functional significance of the ERPs, we further investigated to what extend the brain's response to novelty is affected by differences in emotional valence, arousal, familiarity, or acoustics of the novel sounds, i.e., the emotional vocalizations. To assess the impact of emotional valence or arousal on the MMN, the MMN amplitude was correlated with the rating values for valence and arousal of the explicit rating task for all novel categories. To assess the impact of familiarity, the amplitude of the N1, MMN, P3a, and P3b components were correlated with the grand mean ratings for familiarity. To control for multiple testing, hypothesis-driven Fisher Omnibus tests (Haccou and Melis, 1994) on the *p*-values of the correlation analysis at the four ROIs and ERP time windows for valence, arousal, and familiarity ratings were performed. To investigate whether humans perceive differences between context categories regarding valence, arousal, and familiarity ratings dependent *t*-tests were performed. Early ERP components such as the N1 and MMN are also sensitive to salience of acoustic change between standard and deviant stimuli (e.g., Campbell et al., 2007; Näätänen et al., 2007). Thus, it is important to control for the effect of physical properties especially for early ERPs. To estimate the impact of differences in acoustic properties between standard and novels, the ERP amplitude was correlated with the Euclidian distance between standard and novel sounds, reflecting their acoustic dissimilarity (acoustic dissimilarity index = AD), using the Pearson correlation. The calculation of the Euclidian distance was based on the z-transformed acoustic parameters: Center of gravity, mean peak frequency, percentage of voiced frames, and percentage of call energy. As only 28 participants returned for the rating study, grand averages of ERP components were re-calculated across these participants for the correlation analyses.

In the following, only significant results will be reported. Significant main effects or interactions of the topographical factors only will not be reported. Also, interactions including the factors CON will only be reported if step-down analyses yielded significant effects. All statistical analyses were performed using SPSS 21.

## Results

### Counting task

The mean absolute value of deviations from the true number of targets was 1.95 across blocks (*SD* = 1.51). The value was highest in the second block (2.5; *SD* = 3.06), which contained the highest number of targets (56), and lowest in the last block (1.07; *SD* = 0.69), which contained the lowest number of targets (43), showing that participants were well able to follow and keep up with the counting task from the beginning to the end of the experiment.

### ERPs: effects of species and context

The 4-factorial ANOVA of ERPs responses to the novels (SPECxCONxREGxHEM) revealed main effects of SPEC in all time windows [N1: *F*_(3, 87)_ = 11.38, *p* < 0.001; MMN: *F*_(3, 87)_ = 5.87, *p* ≤ 0.001; P3a: *F*_(3, 87)_ = 7.58, *p* < 0.001; P3b: *F*_(3, 87)_ = 6.72, *p* ≤ 0.002] and of CON for P3a and P3b [P3a: *F*_(3, 87)_ = 7.63, *p* = 0.010; P3b: *F*_(3, 87)_ = 12.33, *p* = 0.001]. In all time windows significant SPECxCON [N1: *F*_(3, 87)_ = 2.92, *p* = 0.038; MMN: *F*_(3, 87)_ = 11.05, *p* < 0.001; P3a: *F*_(3, 87)_ = 6.53, *p* < 0.001; P3b: *F*_(3, 87)_ = 9.30, *p* < 0.001] and SPECxREG interactions were observed [N1: *F*_(3, 87)_ = 3.82, *p* = 0.013; MMN: *F*_(3, 87)_ = 6.47, *p* = 0.001; P3a: *F*_(3, 87)_ = 16.76, *p* < 0.001; P3b: *F*_(3, 87)_ = 13.56, *p* < 0.001]. Further a significant SPECxCONxREG and CONxREG interaction for N1 [SPECxCONxREG: *F*_(3, 87)_ = 7.59, *p* < 0.001; CONxREG: *F*_(1, 29)_ = 49.22, *p* < 0.001] and MMN [SPECxCONxREG: *F*_(3, 87)_ = 6.84, *p* < 0.001; CONxREG: *F*_(1, 29)_ = 25.61, *p* < 0.001] and a significant SPECxCONxHEM interaction for P3b [*F*_(3, 87)_ = 3.91, *p* = 0.013] showed that SPEC greatly influenced novelty processing and that effects of emotional valence and familiarity cannot be interpreted independently of this factor.

In the interest of conciseness and comparability to our behavioral data (Scheumann et al., 2014), we therefore report context effects on the relevant ERPs as step down analyses by species. Whereas, for dog stimuli no significant effect of CON was found in any of the time windows, main effects of CON were detected for human infant, chimpanzee, and tree shrew vocalizations.

Human listeners showed a larger negative response to affiliative than agonistic human infant vocalizations in the MMN time window [main effect CON: *F*_(1, 29)_ = 5.58, *p* = 0.025].

For the chimpanzee vocalizations, there was a significant main effect of CON for the N1 [*F*_(1, 29)_ = 8.10, *p* = 0.008], MMN [*F*_(1, 29)_ = 22.76, *p* < 0.001], and P3b time window [*F*_(1, 29)_ = 4.89, *p* = 0.035], which was qualified for the N1 and MMN time window by a significant CONxREG interaction [N1: *F*_(1, 29)_ = 17.34; MMN: *F*_(1, 29)_ = 4.60, all *p* ≤ 0.041]. The N1 response was larger negative for agonistic vocalizations at the anterior site [LA: *F*_(1, 29)_ = 21.34; RA: *F*_(1, 29)_ = 14.23, all *p* ≤ 0.001], and for the MMN time window the same effect was also confirmed at the posterior scalp site [LA: *F*_(1, 29)_ = 22.40; RA: *F*_(1, 29)_ = 19.65; LP: *F*_(1, 29)_ = 17.65; RP: *F*_(1, 29)_ = 7.63, all *p* ≤ 0.01]. In the P3b time window novel vocalizations led to an enhanced positivity for affiliative vocalizations [main effect CON: *F*_(1, 29)_ = 4.89, *p* = 0.035).

In response to tree shrew vocalizations, a significant CONxREG interaction was observed for the N1 time window [*F*_(1, 29)_ = 45.82, *p* < 0.001], in which the effect of context was larger for agonistic vocalizations over anterior sites [LA: *F*_(1, 29)_ = 20.31; RA: *F*_(1, 29)_ = 20.19; all *p* < 0.001]. This anterior context effect extended to the MMN time window [120–180 ms; CON: *F*_(1, 29)_ = 14.27; *p* < 0.001] where context interacted with REG [*F*_(1, 29)_ = 29.33, *p* < 0.001] and HEMxREG [*F*_(1, 29)_ = 4.98, *p* = 0.034]. Responses to agonistic vocalizations of tree shrews were restricted to anterior sites [LA: *F*_(1, 29)_ = 31.36; RA: *F*_(1, 29)_ = 36.74; all *p* < 0.001]. In the P3a and P3b time windows, there was a main effect of CON [P3a: *F*_(1, 29)_ = 24.00; P3b: *F*_(1, 29)_ = 30.16, all *p* < 0.001], reflecting a stronger positive response to affiliative as compared to agonistic vocalizations.

**Figure 2:**
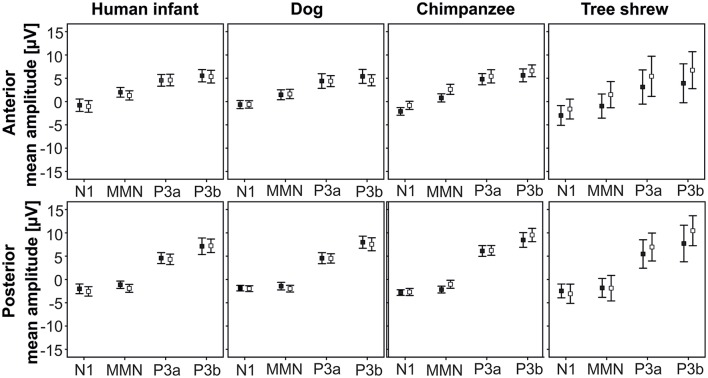
Mean and standard deviations of the ERP amplitudes to agonistic and affiliative vocalizations by species at the anterior and posterior ROIs (pooled for both hemispheres); black square, agonistic vocalizations; white square, affiliative vocalizations.

We found no effects of SPEC on HEM, thus, Figure 2 shows the ERP responses to the two contextual categories by species at the anterior and posterior ROIs.

### Correlation of ERPs with behavioral ratings and acoustic dissimilarity

**Figure 3:**
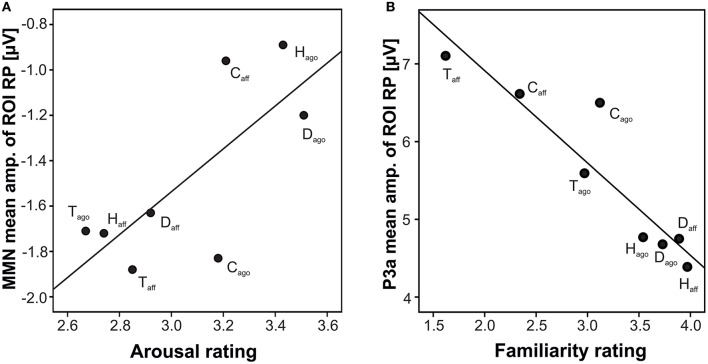
Scatterplot of grand average amplitude of ERP components and behavioral rating per playback category at the right posterior ROIs; **(A)** MMN, arousal; **(B)** P3a, familiarity; H, human infant; D, dog; C, chimpanzee; T, tree shrew; ago, vocalizations recorded in an agonistic context; aff, vocalizations recorded in an affiliative context.

Correlating the behavioral ratings of valence and arousal with the MMN amplitude, a significant correlation of arousal at the right posterior ROI was found (arousal: *r* = 0.742, *N* = 8, *p* = 0.035; Fisher-Omnibustest: χ^2^ = 18.80, df = 8, *p* = 0.016; Figure 3A). Dependent *t*-tests for each species separately revealed significant effects between agonistic and affiliative vocalizations for the valence rating of human infant, dog, and tree shrews [human infant: *t*_(27)_ = −16.54; dog: *t*_(27)_ = −11.83; tree shrew: *t*_(27)_ = 5.37, all *p* < 0.001; see also Scheumann et al., 2014] and for the arousal rating for human infant and dogs [human infant: *t*_(27)_ = 5.56; dog: *t*_(27)_ = 8.93, all *p* < 0.001; Table 1].

**Table 1:** Results of the behavioral ratings (=grand mean for valence, arousal, and familiarity as well as results of the *t*-test comparing the values between context categories), the acoustic dissimilarity index (=Euclidian distance between standard and novel category based on the acoustic parameters per ERP time interval), and ERP effects (=results of the statistical comparison between grand average amplitudes between context categories per species; Aff > Ago: affiliative voice elicits a larger negative or positive amplitude than agonistic voice and Ago > Aff vice versa; ^pA^–correlation between grand average amplitude of the right posterior ROI with arousal; ^pF^–correlation between grand average amplitude of the posterior ROIs with familiarity).

	**Human infant**		**Dog**		**Chimpanzee**		**Tree shrew**	
	**Ago**	**Aff**	**Ago**	**Aff**	**Ago**	**Aff**	**Ago**	**Aff**
**BEHAVIORAL RATINGS**								
**Grand mean valence**	−0.75	1.11	−0.49	0.16	−0.17	−0.24	0.35	−0.21
T	−16.54		−11.83		0.47		5.37	
*p*-value	*p* < 0.001		*p* < 0.001		ns		*p* < 0.001	
**Grand mean arousal**	3.43	2.74	3.51	2.92	3.18	3.21	2.67	2.85
T	5.56		8.93		−0.392		−1.54	
p-value	*p* < 0.001		*p* < 0.001		ns		ns	
**Grand mean familiarity**	3.54	3.97	3.73	3.89	3.12	2.34	2.97	1.62
T	−7.15		5.09		−2.64		7.04	
*p*-value	*p* < 0.001		*p* < 0.001		*p* = 0.014		*p* < 0.001	
**ACOUSTIC DISSIMILARITY INDEX**								
N1	1.71	1.38	1.38	1.15	1.42	3.14	5.04	4.4
MMN	1.25	1.17	1.59	1.17	1.66	3.29	5.42	4.12
P3a	1.28	1.38	2.11	2.06	1.60	3.66	5.20	4.30
P3b	1.29	1.18	2.13	2.87	1.65	3.52	5.23	4.07
**EFFECTS ON ERP COMPONENTS**								
N1	ns		ns		Anterior Ago > Aff		Anterior Ago > Aff	
MMN^pA^	Aff > Ago		ns		Ago > Aff		Anterior Ago > Aff	
P3a^pF^	ns		ns		ns		Aff > Ago	
P3b^pF^	ns		ns		Aff > Ago		Aff > Ago	

Correlating the familiarity scores with the P3a and P3b amplitude at both posterior ROIs resulted in strong negative correlations (P3a: *r* ≤ −0.926, P3b: *r* ≤ −0.906, all *N* = 8, all *p* ≤ 0.002, Figure 3B; Fisher-Omnibustest: χ^2^ ≥ 34.95, *df* = 8, *p* ≤ 0.001). The familiarity scores decreased across species and context of recordings, supporting the fact that vocalizations of human infants and dogs were rated as more familiar than vocalizations of chimpanzees and tree shrews (Table 1). However, a within species analysis further showed that the familiarity scores also significantly differed between affiliative and agonistic vocalizations in all species [human infant: *t*_(27)_ = −7.15; dog: *t*_(27)_ = 5.09; chimpanzee: *t*_(27)_ = −2.64; tree shrew: *t*_(27)_ = 7.04, all *p* < 0.014]. The largest difference for familiarity means was found for tree shrews (Diffmean = 1.35) and chimpanzees (Diffmean = 0.78) compared to human infant (Diffmean = 0.43) and dogs (Diffmean = 0.16).

For the acoustic dissimilarity index, no correlations between AD and absolute amplitudes of ERPs were found, indicating that the acoustic properties of the novel categories did not drive the ERP differences.

## Discussion

To disentangle the impact of evolutionarily retained mechanisms (phylogeny) and experience-driven cognitive processes (familiarity), we investigated the evolution of early and late ERPs for task-irrelevant agonistic and affiliative vocalizations of humans and three animal species.

The animal species varied in their phylogenetic relationship and their familiarity to humans. Comparing ERPs to nonverbal affective human and animal vocalizations revealed that brain responses were strongly affected by the contextual call type. Also a within species analysis showed different effects of the context category on the early and late ERP components across species. These show the typical biphasic novelty response, with early negativities (N1/MMN) and a later positive complex (P3a/P3b) being differentially affected by the properties of the novelty vocalizations. A significant correlation between the MMN amplitude and the arousal rating and a tendency for the valence rating at the posterior site supports the role of the MMN in early emotional processing (e.g., Schirmer and Kotz, 2006). An orthogonal effect of context category on the MMN at posterior sites was found for human infants and chimpanzee vocalizations, but not for dog and tree shrew vocalizations: Human infant's affiliative vocalizations elicited a stronger negativity than agonistic vocalizations whereas for chimpanzee vocalizations the reverse pattern was shown. Concerning familiarity, a significant correlation of the familiarity rating and the P3a may indicate an involuntary attention switch to familiar novels (Friedman et al., 2001; Schirmer and Kotz, 2006; Näätänen et al., 2007), whereas the significant correlation of the familiarity rating and the posterior P3b underlines its role in conscious, cognitive stimulus evaluation (e.g., Cycowicz and Friedman, 1998; Friedman et al., 2001) that is influenced by prior experience. Accordingly, affiliative tree shrew and chimpanzee vocalizations, which were rated as less familiar than the respective agonistic vocalizations and showed the poorest performance when recognizing the emotional valence of the vocalizations, elicited a stronger posterior P3b and for tree shrew vocalizations also a P3a response. This finding strongly supports the influence of familiarity-based processing at this later stage of novelty processing.

Concerning the effects for early emotional processing, we found differences in the brain responses to agonistic and affiliative nonverbal human infant vocalizations in the MMN time window. Infants' laughter evoked a stronger negativity than infants' crying. This finding compares to reports by Seifritz et al. (2003), who showed stronger brain activations in fMRI to infants' laughter than infants' crying in non-parents. The authors concluded that infant cries are of less behavioral relevance to non-parents. Also ERP studies focusing on early emotion responses in task-irrelevant happy sentences or vocalizations reported larger early ERP effects to happy expressions than sad expressions (Paulmann et al., 2013; Pell et al., 2015). Laughing is known to lead to emotional contagion in both human and primates and plays an important role in social interactions (Lundqvist, 1995; Davila Ross et al., 2008, 2011). The contagion character of the infant laughter was also noted in our previous behavioral rating study (Scheumann et al., 2014) where some participants responded to acoustically presented infant laughter's with a smile or laughter (unpublished data). This is in line with Warren et al. (2006), who also found that listening to vocalizations of positive valence and high arousal automatically modulates neural activity engaging the preparation of oro-facial gestures. Thus, the facial expression in response to vocal infant laughter suggest that at least for non-parental male listeners, infant laughter was behaviorally more relevant than infant crying due to its contagious character. The whole head distribution of the MMN effect slightly contradicts an expected fronto-central distribution of this component. However, using passive three-stimulus oddball paradigms showed in some previous work that this early negative response can shift to posterior electrode-sites when participants focus their attention on a target dimension (Oades and Dittmann-Balcar, 1995). Thus, an anterior-posterior shift may result from the counting task utilized here. On the other hand, the posterior MMN effect aligns with the findings by Czigler et al. (2007), who also used a three-tone novelty oddball paradigm. The authors argued that the MMN response “suggests the involvement of a broader neural network in generation of this activity.” This conclusion may also be supported by the results Belin et al. (2008), who argued that “an important component of the limbic system known to be involved in affective processing, the OFC, was activated similarly by valence differences in human and animal vocalizations.” Further, an anterior-posterior shift of early ERPs responses to emotion expressions have also been reported in other studies not necessarily just focusing on early negativities but also positivities. For example, Paulmann et al. (Paulmann and Kotz, 2008; Paulmann et al., 2010) reported a posteriorly distributed positive response to task-irrelevant neutral and emotional stimuli (sad, happy, fearful voice). Further, Jaspers-Fayer et al. (2012) reported an early posterior negativity (time window: 132–156 ms) for emotional compared to neutral stimuli. In the current context, the significant correlation between arousal and the posterior focus on the MMN as well as a trend correlation of the valence rating and the MMN with a more anterior focus calls into question whether emotional valence and arousal processing may be different (Warren et al., 2006; Paulmann et al., 2013). All in all, the majority of studies investigating emotional and neutral stimulus processing did not clearly differentiate whether the reported effects differed as a function of valence or mere arousal (e.g., Schirmer et al., 2005; Czigler et al., 2007; Schirmer and Escoffier, 2010). Concerning the chimpanzee vocalization, it seems unlikely that the posterior early negative effect of context categories relies on arousal only. Participants rated both context categories as similarly arousing in the behavioral rating study (Table 1), and the mean of the arousal rating did not fit well with the correlation (Figure 3A). Thus, further research will have to show to what extent the agonistic and affiliative stimuli may differ in their biological significance to human listeners. We suggest that chimpanzee screams, which are similar in their fundamental frequency contour to human screams may be of higher behavioral importance signaling an urgent threat situation (Arnal et al., 2015).

The significant correlations between the P3b amplitude and the familiarity scores support the functional significance of this late positivity for familiarity (e.g., Mecklinger et al., 1997; Cycowicz and Friedman, 1998; Ylinen et al., 2009). Both chimpanzees and tree shrew vocalizations enhanced a posterior P3b in response to less familiar vocalization types. The missing differences between human infants and dog vocalizations in this late positive ERP response may indicate a ceiling effect as vocalizations of both species are easily recognized and consequently, familiarity scores may differ less between these context categories.

The analysis of the behavioral rating showed significant differences between context categories within most species (for valence: human infant, dog, tree shrew; arousal: human infant, dog; familiarity: all species) and confirm that stimulus properties were distinct enough to perceive differences in valence, arousal, and familiarity between context categories. Nevertheless, the anterior N1, MMN, P3a, and P3b effects did not correlate with valence, arousal, familiarity, or acoustic dissimilarity. This does not imply that these factors do not play a role, but rather that they may be interactive. It is already known that task-irrelevant early negative ERP responses play a significant role in deviance detection (e.g., Campbell et al., 2007; Näätänen et al., 2007). Given that all vocalizations used in the current experiment were naturally induced and acoustically different between species and context categories, it is apparent that acoustic differences may have influenced early ERP responses. We addressed this issue by calculating the acoustic dissimilarity index between standards and novels as a measure for differences in the acoustical properties. As we found no correlation between acoustic dissimilarity and the early ERP responses, we can at least rule out that ERP amplitude differences between context categories can be explained by a simple effect of acoustic differences between standards and novels. Further, novel categories that were acoustically less similar to standards did not evoke larger ERP responses than novel categories that were more similar. Studies on early emotional processing and familiarity also showed effects on the above mentioned ERP components (e.g., familiarity: Thierry et al., 2003; Jacobsen et al., 2005; Kirmse et al., 2009; emotion: Paulmann and Kotz, 2008; Liu et al., 2012; Jiang et al., 2014) and suggest that acoustic variation especially in the early ERPs does not exclusively reflect deviant detection of acoustic properties. Moreover, Jiang et al. (2014) showed an additive effect of emotion and acoustic properties in deviant ERP responses. Such cumulative effects make it difficult to interpret the role of valence, arousal, familiarity, and acoustic properties without additional experiments controlling for each factor, respectively.

In summary, the current results indicate a strong influence of stimulus familiarity on the P3a and P3b in a novelty oddball paradigm. We further found indications of early emotional processing, potentially independent of attention in the MMN time window that cannot be easily explained by differences in familiarity or acoustic stimulus properties and may therefore reflect an evolutionary retained mechanism allowing for the rapid evaluation of emotional content across related species. In the future, it could be helpful to conduct ERP experiments for each species. Thus, familiarity differences across species may shift the deviant detection to familiarity detection and thereby limit the detection of emotional salience.

## Author contributions

MS designed the experiment, recorded, prepared and analyzed the acoustic stimuli, performed and analyzed the ERP experiments, prepared, performed and analyzed the behavioral rating experiments and wrote the manuscript; AH designed the experiment, prepared, programmed and analyzed the ERP experiment, prepared and programmed the behavioral rating experiment and wrote the manuscript; SK and EZ designed the experiment and wrote/edited the manuscript.

### Conflict of interest statement

The authors declare that the research was conducted in the absence of any commercial or financial relationships that could be construed as a potential conflict of interest.

## References

[B1] Arnal L. H. Flinker A. Kleinschmidt A. Giraud A. L. Poeppel D. (2015). Human Screams occupy a privileged niche in the communication soundscape. Curr. Biol. 25, 2051–2056. 10.1016/j.cub.2015.06.043 26190070 PMC4562283

[B2] Bastian A. Schmidt S. (2008). Affect cues in vocalizations of the bat, *Megaderma lyra*, during agonistic interactions. J. Acoust. Soc. Am. 124, 598–608. 10.1121/1.2924123 18647002

[B3] Belin P. Fecteau S. Charest I. Nicastro N. Hauser M. D. Armony J. L. (2008). Human cerebral response to animal affective vocalizations. Proc. R. Soc. B Biol. Sci. 275, 473–481. 10.1098/rspb.2007.1460 18077254 PMC2596811

[B4] Boersma P. (2001). Praat, a system for doing phonetics by computer. Glot Int. 5, 341–345.

[B5] Bradley M. M. Lang P. J. (1994). Measuring emotion: the self-assessment manikin and the semantic differential. J. Behav. Ther. Exp. Psychiatry 25, 49–59. 10.1016/0005-7916(94)90063-9 7962581

[B6] Campbell T. Winkler I. Kujala T. (2007). N1 and the mismatch negativity are spatiotemporally distinct ERP components: disruption of immediate memory by auditory distraction can be related to N1. Psychophysiology 44, 530–540. 10.1111/j.1469-8986.2007.00529.x 17532805

[B7] Cycowicz Y. M. Friedman D. (1998). Effect of sound familiarity on the event-related potentials elicited by novel environmental sounds. Brain Cogn. 36, 30–51. 10.1006/brcg.1997.0955 9500881

[B8] Czigler I. Cox T. J. Gyimesi K. Horvath J. (2007). Event-related potential study to aversive auditory stimuli. Neurosci. Lett. 420, 251–256. 10.1016/j.neulet.2007.05.007 17556101

[B9] Darwin C. (1872). The Expression of Emotion in Man and Animals. London: John Murray.

[B10] Davila Ross M. Allcock B. Thomas C. Bard K. A. (2011). Aping expressions? Chimpanzees produce distinct laugh types when responding to laughter of others. Emotion 11, 1013–1020. 10.1037/a0022594 21355640

[B11] Davila Ross M. Menzler S. Zimmermann E. (2008). Rapid facial mimicry in orangutan play. Biol. Lett. 4, 27–30. 10.1098/rsbl.2007.0535 18077238 PMC2412946

[B12] Davila Ross M. Owren M. J. Zimmermann E. (2009). Reconstructing the evolution of laughter in great apes and humans. Curr. Biol. 19, 1106–1111. 10.1016/j.cub.2009.05.028 19500987

[B13] Ehret G. (2006). Common rules of communication sound perception, in Behavior and Neurodynamics for Auditory Communication, eds Kanwal J. Ehret G. (Cambridge: Cambridge University Press), 85–114.

[B14] Fecteau S. Armony J. L. Joanette Y. Belin P. (2005). Judgment of emotional nonlinguistic vocalizations: age-related differences. Appl. Neuropsychol. 12, 40–48. 10.1207/s15324826an1201_7 15788222

[B15] Field A. (2009). Discovering Statistics using SPSS. London: Sage Publications.

[B16] Flom R. Whipple H. Hyde D. (2009). Infants' intermodal perception of canine (*Canis familaris*) facial expressions and vocalizations. Dev. Psychol. 45, 1143–1151. 10.1037/a0015367 19586184

[B17] Friedman D. Cycowicz Y. M. Gaeta H. (2001). The novelty P3: an event-related brain potential (ERP) sign of the brain's evaluation of novelty. Neurosci. Biobehav. Rev. 25, 355–373. 10.1016/S0149-7634(01)00019-7 11445140

[B18] Gogoleva S. S. Volodin I. A. Volodina E. V. Kharlamova A. V. Trut L. N. (2010). Sign and strength of emotional arousal: vocal correlates of positive and negative attitudes to humans in silver foxes (*Vulpes vulpes*). Behaviour 147, 1713–1736. 10.1163/000579510X528242

[B19] Haccou P. Melis E. (1994). Statistical Analysis of Behavioural Data. New York, NY: Oxford University Press.

[B20] Hammerschmidt K. Jürgens U. (2007). Acoustical correlates of affective prosody. J. Voice 21, 531–540. 10.1016/j.jvoice.2006.03.002 16647247

[B21] Ho H. T. Schroger E. Kotz S. A. (2015). Selective attention modulates early human evoked potentials during emotional face-voice processing. J. Cogn. Neurosci. 27, 798–818. 10.1162/jocn_a_00734 25269113

[B22] Jacobsen T. Schroger E. Winkler I. Horvath J. (2005). Familiarity affects the processing of task-irrelevant auditory deviance. J. Cogn. Neurosci. 17, 1704–1713. 10.1162/089892905774589262 16269107

[B23] Jaspers-Fayer F. Ertl M. Leicht G. Leupelt A. Mulert C. (2012). Single-trial EEG-fMRI coupling of the emotional auditory early posterior negativity. Neuroimage 62, 1807–1814. 10.1016/j.neuroimage.2012.05.018 22584235

[B24] Jessen S. Kotz S. A. (2011). The temporal dynamics of processing emotions from vocal, facial, and bodily expressions. Neuroimage 58, 665–674. 10.1016/j.neuroimage.2011.06.035 21718792

[B25] Jiang A. Yang J. Yang Y. (2014). MMN responses during implicit processing of changes in emotional prosody: an ERP study using Chinese pseudo-syllables. Cogn. Neurodyn. 8, 499–508. 10.1007/s11571-014-9303-3 26396648 PMC4571648

[B26] Juslin P. N. Laukka P. (2003). Communication of emotions in vocal expression and music performance: different channels, same code? Psychol. Bull. 129, 770–814. 10.1037/0033-2909.129.5.770 12956543

[B27] Kirmse U. Jacobsen T. Schroger E. (2009). Familiarity affects environmental sound processing outside the focus of attention: an event-related potential study. Clin. Neurophys. 120, 887–896. 10.1016/j.clinph.2009.02.159 19345610

[B28] Kirmse U. Schroger E. Jacobsen T. (2012). Familiarity of environmental sounds is used to establish auditory rules. Neuroreport 23, 320–324. 10.1097/WNR.0b013e328351760b 22410549

[B29] Kokinous J. Kotz S. A. Tavano A. Schroger E. (2015). The role of emotion in dynamic audiovisual integration of faces and voices. Soc. Cogn. Affect. Neurosci. 10, 713–720. 10.1093/scan/nsu105 25147273 PMC4420746

[B30] Linnankoski I. Laakso M. Aulanko R. Leinonen L. (1994). Recognition of emotions in macaque vocalizations by children and adults. Lang. Commun. 14, 183–192. 10.1016/0271-5309(94)90012-4

[B31] Liu P. Rigoulot S. Pell M. D. (2015). Cultural differences in on-line sensitivity to emotional voices: comparing East and West. Front. Hum. Neurosci. 9:311. 10.3389/fnhum.2015.00311 26074808 PMC4448034

[B32] Liu T. Pinheiro A. P. Deng G. Nestor P. G. McCarley R. W. Niznikiewicz M. A. (2012). Electrophysiological insights into processing nonverbal emotional vocalizations. Neuroreport 23, 108–112. 10.1097/WNR.0b013e32834ea757 22134115

[B33] Lundqvist L.-O. (1995). Facial EMG reactions to facial expression: a case facial emotional contagion? Scand. J. Psychol. 36, 130–141. 10.1111/j.1467-9450.1995.tb00974.x 7644897

[B34] Mecklinger A. Opitz B. Friederici A. D. (1997). Semantic aspects of novelty detection in humans. Neurosci. Lett. 235, 65–68. 10.1016/S0304-3940(97)00712-X 9389597

[B35] Molnár C. Pongrácz P. Dóka A. Miklósi A. (2006). Can humans discriminate between dogs on the base of the acoustic parameters of barks? Behav. Process. 73, 76–83. 10.1016/j.beproc.2006.03.014 16678361

[B36] Molnár C. Pongrácz P. Miklósi A. (2010). Seeing with ears: sightless humans' perception of dog bark provides a test for structural rules in vocal communication. Q. J. Exp. Psychol. 63, 1004–1013. 10.1080/17470210903168243 19760535

[B37] Morton E. S. (1977). On the occurrence and significance of motivation-structural rules in some bird and mammal sounds. Am. Nat. 111, 855–869. 10.1086/283219

[B38] Näätänen R. Paavilainen P. Rinne T. Alho K. (2007). The mismatch negativity (MMN) in basic research of central auditory processing: a review. Clin. Neurophys. 118, 2544–2590. 10.1016/j.clinph.2007.04.026 17931964

[B39] Nicastro N. Owren M. J. (2003). Classification of domestic cat (*Felis catus*) vocalizations by naive and experienced human listeners. J. Comp. Psychol. 117, 44–52. 10.1037/0735-7036.117.1.44 12735363

[B40] Oades R. D. Dittmann-Balcar A. (1995). Mismatch negativity (MMN) is altered by directing attention. Neuroreport 6, 1187–1190. 10.1097/00001756-199505300-00028 7662904

[B41] Oldfield R. C. (1971). The assessment and analysis of handedness: the Edinburgh inventory. Neuropsychologia 9, 97–113. 10.1016/0028-3932(71)90067-4 5146491

[B42] Paulmann S. Bleichner M. Kotz S. A. (2013). Valence, arousal, and task effects in emotional prosody processing. Front. Psychol. 4:345. 10.3389/fpsyg.2013.00345 23801973 PMC3689289

[B43] Paulmann S. Kotz S. A. (2008). Early emotional prosody perception based on different speaker voices. Neuroreport 19, 209–213. 10.1097/WNR.0b013e3282f454db 18185110

[B44] Paulmann S. Seifert S. Kotz S. A. (2010). Orbito-frontal lesions cause impairment during late but not early emotional prosodic processing. Soc. Neurosci. 5, 59–75. 10.1080/17470910903135668 19658025

[B45] Pell M. D. Monetta L. Paulmann S. Kotz S. A. (2009a). Recognizing emotions in a foreign language. J. Nonverb. Behav. 33, 107–120. 10.1007/s10919-008-0065-7

[B46] Pell M. D. Paulmann S. Dara C. Alasseri A. Kotz S. A. (2009b). Factors in the recognition of vocally expressed emotions: a comparison of four languages. J. Phonetics 37, 417–435. 10.1016/j.wocn.2009.07.005

[B47] Pell M. D. Rothermich K. Liu P. Paulmann S. Sethi S. Rigoulot S. (2015). Preferential decoding of emotion from human non-linguistic vocalizations versus speech prosody. Biol. Psychol. 111, 14–25. 10.1016/j.biopsycho.2015.08.008 26307467

[B48] Pfeifer E. Novagk R. Maeβ B. (1995). Software for EEG/ERP Evaluation. Leipzig: Max Planck Institute of Cognitive Neuroscience.

[B49] Pongrácz P. Molnár C. Dóka A. Miklósi A. (2011). Do children unterstand man's best friend? Classification of dog barks by pre-adolescents and adults. Appl. Anim. Behav. Sci. 135, 95–102. 10.1016/j.applanim.2011.09.005

[B50] Pongrácz P. Molnár C. Miklósi A. (2006). Acoustic parameters of dog barks carry emotional information for humans. Appl. Anim. Behav. Sci. 100, 228–240. 10.1016/j.applanim.2005.12.004

[B51] Pongrácz P. Molnár C. Miklósi A. (2010). Barking in family dogs: an ethological approach. Vet. J. 183, 141–147. 10.1016/j.tvjl.2008.12.010 19181546

[B52] Pongrácz P. Molnár C. Miklósi A. Csányi V. (2005). Human listeners are able to classify dog (*Canis familiaris*) barks recorded in different situations. J. Comp. Psychol. 119, 136–144. 10.1037/0735-7036.119.2.136 15982157

[B53] Sander K. Frome Y. Scheich H. (2007). FMRI activations of amygdala, cingulate cortex, and auditory cortex by infant laughing and crying. Hum. Brain Mapp. 28, 1007–1022. 10.1002/hbm.20333 17358020 PMC6871318

[B54] Sauter D. A. Eimer M. (2010). Rapid detection of emotion from human vocalizations. J. Cogn. Neurosci. 22, 474–481. 10.1162/jocn.2009.21215 19302002

[B55] Sauter D. A. Eisner F. Ekman P. Scott S. K. (2010). Cross-cultural recognition of basic emotions through nonverbal emotional vocalizations. Proc. Natl. Acad. Sci. U.S.A. 107, 2408–2412. 10.1073/pnas.0908239106 20133790 PMC2823868

[B56] Schehka S. Zimmermann E. (2009). Acoustic features to arousal and identity in disturbance calls of tree shrews (*Tupaia belangeri*). Behav. Brain Res. 203, 223–231. 10.1016/j.bbr.2009.05.007 19445967

[B57] Scherer K. R. Banse R. Wallbott H. G. (2001). Emotion inferences from vocal expression correlate across language and culture. Cross Cult. Psychol. 32, 76–92. 10.1177/0022022101032001009

[B58] Scheumann M. Hasting A. S. Kotz S. A. Zimmermann E. (2014). The voice of emotion across species: how do human listeners recognize animals' affective states? PLoS ONE 9:e91192. 10.1371/journal.pone.0091192 24621604 PMC3951321

[B59] Scheumann M. Roser A. E. Konerding W. Bleich E. Hedrich H. J. Zimmermann E. (2012). Vocal correlates of sender-identity and arousal in the isolation calls of domestic kitten (*Felis silvestris catus*). Front. Zool. 9:36. 10.1186/1742-9994-9-36 23259698 PMC3551667

[B60] Scheumann M. Zimmermann E. Deichsel G. (2007). Context-specific calls signal infants' needs in a strepsirrhine primate, the gray mouse lemur (*Microcebus murinus*). Dev. Psychobiol. 49, 708–718. 10.1002/dev.20234 17943980

[B61] Schirmer A. Escoffier N. (2010). Emotional MMN: anxiety and heart rate correlate with the ERP signature for auditory change detection. Clin. Neurophys. 121, 53–59. 10.1016/j.clinph.2009.09.029 19896894

[B62] Schirmer A. Kotz S. A. (2006). Beyond the right hemisphere: brain mechanisms mediating vocal emotional processing. Trends Cogn. Sci. 10, 24–30. 10.1016/j.tics.2005.11.009 16321562

[B63] Schirmer A. Striano T. Friederici A. (2005). Sex differences in the preattentive processing of vocal emotional expression. Neuroreport 16 635–639. 10.1097/00001756-200504250-00024 15812323

[B64] Schirmer A. Zysset S. Kotz S. Von Cramon Y. (2004). Gender differences in the activation of inferior frontal cortex during emotional speech perception. Neuroimage 21, 1114–1123. 10.1016/j.neuroimage.2003.10.048 15006679

[B65] Seifritz E. Esposito F. Neuhoff J. G. Luthi A. Mustovic H. Dammann G.. (2003). Differential sex-independent amygdala response to infant crying and laughing in parents versus nonparents. Biol. Psychiatry 54, 1367–1375. 10.1016/S0006-3223(03)00697-8 14675800

[B66] Shahin A. Bosnyak D. J. Trainor L. J. Roberts L. E. (2003). Enhancement of neuroplastic P2 and N1c auditory evoked potentials in musicians. J. Neurosci. 23, 5545–5552. 12843255 10.1523/JNEUROSCI.23-13-05545.2003 PMC6741225

[B67] Shahin A. Roberts L. E. Trainor L. J. (2004). Enhancement of auditory cortical development by musical experience in children. Neuroreport 15, 1917–1921. 10.1097/00001756-200408260-00017 15305137

[B68] Sharbrough F. Chatrian G.-E. Lesser R. Lüders H. Nuwer M. Picton T. (1991). American electroencephalographic society guidelines for standard electrode position nomenclature. J. Clin. Neurophysiol. 8, 200–202. 10.197/00004691-1991004000-00007 2050819

[B69] Soltis J. Leong K. Savage A. (2005). African elephant vocal communication II: rumble variation reflects the individual identity and emotional state of callers. Anim. Behav. 70, 589–599. 10.1016/j.anbehav.2004.11.016

[B70] Tallet C. Špinka M. Maruščáková I. Šimeček P. (2010). Human perception of vocalizations of domestic piglets and modulation by experience with domestic pigs (*Sus scrofa*). J. Comp. Psychol. 124, 81–91. 10.1037/a0017354 20175599

[B71] Taylor A. Reby D. McComb K. (2009). Context-related variation in the vocal growling behaviour of the domestic dog (*Canis familiaris*). Ethology 115, 905–915. 10.1111/j.1439-0310.2009.01681.x

[B72] Thierry G. Vihman M. Roberts M. (2003). Familiar words capture the attention of 11-month-olds in less than 250 ms. Neuroreport 14, 2307–2310. 10.1097/00001756-200312190-00004 14663181

[B73] Vettin J. Todt D. (2005). Human laughter, social play, and play vocalizations of non-human primates: an evolutionary approach. Behaviour 142, 217–240. 10.1163/1568539053627640

[B74] Warren J. E. Sauter D. A. Eisner F. Wiland J. Dresner M. A. Wise R. J. S.. (2006). Positive emotions preferentially engage an auditory-motor “mirror” system. J. Neurosci. 26, 13067–13075. 10.1523/JNEUROSCI.3907-06.2006 17167096 PMC6674947

[B75] Ylinen S. Strelnikov K. Huotilainen M. Naatanen R. (2009). Effects of prosodic familiarity on the automatic processing of words in the human brain. Int. J. Psychophysiol. 73, 362–368. 10.1016/j.ijpsycho.2009.05.013 19482051

[B76] Zeskind P. S. Marshall T. R. (1988). The relation between variations in pitch and maternal perceptions of infant crying. Child Dev. 59, 193–196. 10.2307/1130401

[B77] Zimmermann E. (2010). Vocal expression of emotion in a nocturnal prosimian primate group, mouse lemurs, in Handbook of Mammalian Vocalization: An Integrative Neuroscience Approach, ed Brudzynski S. M. (Oxford: Academic Press), 215–225.

[B78] Zimmermann E. Leliveld L. M. C. Schehka S. (2013). Toward the evolutionary roots of affective prosody in human acoustic communication: a comparative approach to mammalian voices, in Evolution of Emotional Communication: From Sound in Nonhuman Mammals to Speech and Music in Man, eds Altenmüller E. Schmidt S. Zimmermann E. (Oxford: Oxford University Press), 116–132.

