# Supplementary material for: Human Novelty Response to Emotional Animal Vocalizations: Effects of Phylogeny and Familiarity
Source: Front Behav Neurosci. 2017 Oct 24;11:204. doi: 10.3389/fnbeh.2017.00204 (PMC5660701; doi:10.3389/fnbeh.2017.00204)
Supplement: Supplementary file 1 [file DataSheet1.docx]

Supplementary Material

**Human novelty response to emotional animal vocalizations: Effects of phylogeny and familiarity**

**Marina Scheumann ^+1^, Anna S. Hasting ^+2,3^, Elke Zimmermann^1^ & Sonja A. Kotz^2,4*^**

^+^ contributed equally to this work

*** Correspondence:** Sonja Kotz, [kotz@cbs.mpg.de](mailto:kotz@cbs.mpg.de)

**Supplementary 1:** Detailed context description of acoustic stimuli

In general, affiliative acoustic stimuli were recorded in context where vocalisations were used to maintain a current interaction whereas agonistic acoustic stimuli were recorded in context where vocalisations were used to interrupt or to change a current interaction (for further details see also Scheumann et al., 2014)

| Affiliative human infant stimuli: | Infant laughter’s were recorded during tickling or playing sessions from 1½ year old infants. |
| --- | --- |
| Affiliative dog stimuli: | Dog barks were recorded during playing with either other dogs (16 calls) or a human (8 calls). |
| Affiliative chimpanzee stimuli: | Chimpanzee laughters were recorded during tickling sessions from five infants and one subadult chimpanzee. |
| Affiliative tree shrew stimuli: | Tree shrew rhythmic clicking was recorded during male-female  non-agonistic mating interactions. |
| Affiliative human infant stimuli: | Infant cries or screams were recorded from 1 ½ year old infants when the mother forbids the infant something or another infant took a toy away from the sender. |
| Agonistic chimpanzee stimuli: | Chimpanzee screams were recorded during aggressive interactions between adult chimpanzees including physical conflicts. |
| Agonistic dog stimuli: | Dog barks and growls were recorded during aggressive conflicts between dogs (16 calls) or when a strange person approached the dog (8 calls). |
| Agonistic tree shrew stimuli: | Tree shrew Squeaks were recorded when the sender was chasing around by a conspecific. |

**Supplementary 2:** Preparation of acoustic stimuli

Based on the audio recordings of the respective context, we selected 24 calls of good signal-to-noise ratio per species and context to prepare the acoustic stimuli for the ERP experiment.

To standardize the duration of the stimuli, we selected natural calls or call sequences matching approximately 1 second duration. Vocalisations were cut using the software SIGNAL 3.1 (Engineering Design, Berkeley, California, U.S.A.). Thereby, the acoustic stimuli started immediately with the respective vocalisation. Thus, the vocalizations were cut at the start point at zero-point crossing of the amplitude to avoid clicks.

To keep the sound intensity equal across playback stimuli, we used PRAAT ([www.praat.org](http://www.praat.org); Boersma, 2001) to normalize the sound intensity to 60 dB.

**Supplementary 3:** Overview about the acoustic parameters for the respective analysis windows

Following acoustic parameters were measured using PRAAT from the start of the stimuli to the onset of the respective ERP component: Center of gravity (=mean frequency of the spectrum in Hz; CoG), peak frequency (=frequency with maximum energy in Hz; PEAK), percentage of voiced frames (=no. of voiced frames of a call divided by the total number of frames in %; %VOI) and percentage of call energy (=call duration divided by the total duration of the analysed window in %; %ENERGY). Means for each playback category of the novels and standard and deviant are listed in Table S1.

Sonograms of the stimuli can be viewed in Scheumann et al. (2014; Figure 1, open access).

Table S1: Means of acoustic parameters for novels (NOV = novel, H – human infant, D – dog, C – chimpanzee, T – tree shrew, ago – vocalizations recorded in an agonistic context, aff – vocalizations recorded in an affiliative context) and the standard (STD) and target (TAR) stimuli for the analysed time windows of the respective ERP component

| Analysed Time Window | Playback category | %VOI | PEAK | %ENERGY | CoG |
| --- | --- | --- | --- | --- | --- |
| 0 - 70 ms  (N1) | NOV - H_ago_ | 80.35 | 1126.01 | 95.60 | 1445.10 |
|  | NOV - H_aff_ | 86.90 | 857.10 | 96.07 | 1058.18 |
|  | NOV - D_ago_ | 47.61 | 729.82 | 100.00 | 922.03 |
|  | NOV - D_aff_ | 58.92 | 836.98 | 100.00 | 1152.03 |
|  | NOV - C_ago_ | 95.83 | 1729.45 | 100.00 | 1925.75 |
|  | NOV - C_aff_ | 0.00 | 1703.94 | 97.44 | 2262.97 |
|  | NOV - T_ago_ | 85.71 | 4085.09 | 92.86 | 4913.35 |
|  | NOV - T_aff_ | 0.00 | 1611.34 | 92.38 | 4031.24 |
|  | STD | 100.00 | 600.00 | 100.00 | 600.00 |
|  | TAR | 100.00 | 660.00 | 100.00 | 660.00 |
| 0 – 120 ms  (N2/MMN) | NOV - H_ago_ | 79.86 | 1157.63 | 91.53 | 1402.72 |
|  | NOV - H_aff_ | 78.13 | 898.79 | 90.00 | 1104.51 |
|  | NOV - D_ago_ | 41.33 | 776.95 | 99.44 | 932.04 |
|  | NOV - D_aff_ | 61.11 | 931.50 | 99.48 | 1199.31 |
|  | NOV - C_ago_ | 95.83 | 1796.68 | 100.00 | 2013.42 |
|  | NOV - C_aff_ | 0.00 | 1545.12 | 86.77 | 2202.41 |
|  | NOV - T_ago_ | 65.28 | 3580.49 | 69.10 | 5134.26 |
|  | NOV - T_aff_ | 0.00 | 1208.63 | 75.80 | 3801.63 |
|  | STD | 100.00 | 600.00 | 100.00 | 600.00 |
|  | TAR | 100.00 | 660.00 | 100.00 | 660.00 |
| 0 – 210 ms  (P3a) | NOV - H_ago_ | 78.38 | 1183.36 | 89.66 | 1506.48 |
|  | NOV - H_aff_ | 72.02 | 860.99 | 85.50 | 1108.86 |
|  | NOV - D_ago_ | 32.93 | 755.20 | 84.60 | 956.96 |
|  | NOV - D_aff_ | 46.63 | 797.24 | 80.52 | 1179.72 |
|  | NOV - C_ago_ | 97.42 | 1842.71 | 100.00 | 2021.31 |
|  | NOV - C_aff_ | 0.00 | 1509.21 | 71.85 | 2299.44 |
|  | NOV - T_ago_ | 64.10 | 3804.48 | 70.04 | 5351.31 |
|  | NOV - T_aff_ | 0.00 | 921.29 | 62.64 | 3501.20 |
|  | STD | 100.00 | 600.00 | 100.00 | 600.00 |
|  | TAR | 100.00 | 660.00 | 100.00 | 660.00 |
| 0 – 290 ms  (P3b) | NOV - H_ago_ | 76.45 | 1207.59 | 88.36 | 1532.76 |
|  | NOV - H_aff_ | 74.86 | 867.72 | 85.33 | 1161.70 |
|  | NOV - D_ago_ | 36.20 | 750.89 | 77.67 | 975.54 |
|  | NOV - D_aff_ | 34.05 | 701.99 | 61.29 | 1174.45 |
|  | NOV - C_ago_ | 97.57 | 1852.88 | 100.00 | 2033.65 |
|  | NOV - C_aff_ | 0.00 | 1411.42 | 67.60 | 2305.20 |
|  | NOV - T_ago_ | 55.18 | 3659.31 | 62.27 | 5404.21 |
|  | NOV - T_aff_ | 0.00 | 929.93 | 58.05 | 3477.92 |
|  | STD | 100.00 | 600.00 | 100.00 | 600.00 |
|  | TAR | 100.00 | 660.00 | 100.00 | 660.00 |
